# Partial Agonist, Telmisartan, Maintains PPARγ Serine 112 Phosphorylation, and Does Not Affect Osteoblast Differentiation and Bone Mass

**DOI:** 10.1371/journal.pone.0096323

**Published:** 2014-05-08

**Authors:** Vipula Kolli, Lance A. Stechschulte, Abigail R. Dowling, Sima Rahman, Piotr J. Czernik, Beata Lecka-Czernik

**Affiliations:** 1 Department of Orthopaedic Surgery, University of Toledo College of Medicine, Toledo, Ohio, United States of America; 2 Department of Physiology and Pharmacology, University of Toledo College of Medicine, Toledo, Ohio, United States of America; 3 Center for Diabetes and Endocrine Research, University of Toledo College of Medicine, Toledo, Ohio, United States of America; Uniformed Services University of the Health Sciences, United States of America

## Abstract

Peroxisome proliferator activated receptor gamma (PPARγ) controls both glucose metabolism and an allocation of marrow mesenchymal stem cells (MSCs) toward osteoblast and adipocyte lineages. Its activity is determined by interaction with a ligand which directs posttranscriptional modifications of PPARγ protein including dephosphorylation of Ser112 and Ser273, which results in acquiring of pro-adipocytic and insulin-sensitizing activities, respectively. PPARγ full agonist TZD rosiglitazone (ROSI) decreases phosphorylation of both Ser112 and Ser273 and its prolonged use causes bone loss in part due to diversion of MSCs differentiation from osteoblastic toward adipocytic lineage. Telmisartan (TEL), an anti-hypertensive drug from the class of angiotensin receptor blockers, also acts as a partial PPARγ agonist with insulin-sensitizing and a weak pro-adipocytic activity. TEL decreased ^S273^pPPARγ and did not affect ^S112^pPPARγ levels in a model of marrow MSC differentiation, U-33/γ2 cells. In contrast to ROSI, TEL did not affect osteoblast phenotype and actively blocked ROSI-induced anti-osteoblastic activity and dephosphorylation of ^S112^pPPARγ. The effect of TEL on bone was tested side-by-side with ROSI. In contrast to ROSI, TEL administration did not affect bone mass and bone biomechanical properties measured by micro-indentation method and did not induce fat accumulation in bone, and it partially protected from ROSI-induced bone loss. In addition, TEL induced “browning” of epididymal white adipose tissue marked by increased expression of UCP1, FoxC2, Wnt10b and IGFBP2 and increased overall energy expenditure. These studies point to the complexity of mechanisms by which PPARγ acquires anti-osteoblastic and pro-adipocytic activities and suggest an importance of Ser112 phosphorylation status as being a part of the mechanism regulating this process. These studies showed that TEL acts as a full PPARγ agonist for insulin-sensitizing activity and as a partial agonist/partial antagonist for pro-adipocytic and anti-osteoblastic activities. They also suggest a relationship between PPARγ fat “browning” activity and a lack of anti-osteoblastic activity.

## Introduction

Peroxisome proliferator activated receptor gamma (PPARγ) belongs to a family of DNA-binding nuclear receptors and functions as an adipocyte-specific transcription factor and a key regulator of cellular insulin sensitivity [1]. PPARγ also controls bone mass by regulating commitment of mesenchymal stem cells (MSCs) toward osteoblasts and adipocytes [2], [3]. When activated with full agonists, e.g. anti-diabetic TZDs rosiglitazone (ROSI) and pioglitazone, PPARγ suppresses osteoblasts and promotes adipocytes development, and enhances support for osteoclast development [2], [4]–[6]. Prolonged use of TZDs leads to bone loss and increases occurrence of fractures, especially in older women (reviewed in [7]). As shown in mice, the deleterious effect of TZDs on bone also includes suppression of new bone formation and accumulation of large quantities of fat at the bone healing site [8], [9], suggesting a possibility of significant orthopaedic complications in fracture healing of diabetic patients on therapy with full PPARγ agonists.

Upon ligand binding, PPARγ protein acquires a spectrum of posttranscriptional modifications (PTMs), which determine its specific activities. PTMs include serine phosphorylation, acetylation and lysine sumoylation [10]. Dephosphorylation of Ser273 is essential for acquiring insulin-sensitizing activity [11], whereas dephosphorylation of Ser112 is essential for acquiring transcriptional pro-adipocytic activity by PPARγ [12], [13]. PPARγ pro-adipocytic activity includes directing adipocytes to acquire a phenotype regulating either energy storage through lipogenesis or energy dissipation through lipolysis. Customarily, fat depots involved in energy storage are named white adipose tissue (WAT), whereas depots involved in energy production, which requires large numbers of mitochondria, are called brown adipose tissue (BAT) [14]. Recently, the third type of adipocytes has been identified and named “beige” or “brite” because, while being located within WAT depots and perhaps originating from the same progenitors as white adipocytes, they may acquire BAT function for energy dissipation in response to cold or pharmacologic stimuli [15], [16].

Telmisartan (TEL) belongs to a family of anti-hypertensive drugs, known as angiotensin 2 receptor blockers (ARBs), which target renin-angiotensin system (RAS) regulating body fluid, electrolyte balance and blood pressure. RAS is recognized as contributing to the development of osteoporosis independently of hypertension [17]–[19], and a blockage of this system either at the level of angiotensin enzyme inhibitor (ACEI) or at the level of angiotensin receptors proved to be beneficial for bone [20], [21]. Beside its anti-hypertensive activity, TEL has a unique ability to bind and activate PPARγ [22], [23] and has a beneficial effects on insulin sensitivity in humans [24]–[26] and rodents [27]. As compared to full agonists, pioglitazone and ROSI, TEL binds PPARγ in a different fashion which results in a distinct pattern of cofactors recruitment and different pharmacological effects [28]. It has been reported that TEL alleviates ROSI-induced bone loss in ovariectomized rats; however the mechanism for this effect has not been provided [29]. The aim of this study was to characterize TEL as PPARγ agonist regulating its osteoblastic and adipocytic activities.

TEL-mediated PPARγ activities were tested *in vitro* in a model of marrow MSCs differentiation and its effect on bone and energy metabolism was tested in two murine models of Type 2 diabetes, yellow agouti A^vy^/a mice and C57BL/6 mice with diet-induced obesity (DIO). We have found that in contrast to full agonist ROSI, TEL blocks PPARγ anti-osteoblastic activity while inducing insulin-sensitizing activity. Moreover, TEL induced “beiging” of WAT and increased energy expenditure. These effects of TEL correlated with decreased levels of Ser273 phosphorylation and unchanged levels of Ser112 phosphorylation of PPARγ protein.

## Materials and Methods

### Reagents

Reagents were obtained from the following sources: MEM-α medium (Invitrogen, Carlsbad, CA), fetal bovine serum (Hyclone, Logan, UT), G418 (Sigma-Aldrich, St. Louis, MO), rosiglitazone (Tularik, Inc., San Francisco, CA), Avandia (rosiglitazone maleate) (GlaxoSmithKline, King of Prusia, PA), telmisartan and losartan (Sigma-Aldrich), Micardis (telmisartan) (Boehringer Ingelheim, Ridgefield, CT), Power SYBR Green PCR Master Mix (Applied Biosystems, Carlsbad, CA), and Cell Titer 96 AQ_ueous_ Non-Radioactive Cell Proliferation Assay (Promega, Madison, WI).). Antibody against PPARγ (sc-7273) was obtained from Santa Cruz Biotechnologies (Santa Cruz, CA). Ser-112 Phospho-PPARγ2 antibody was purchased from Abcam (Abcam PLC, Cambridge, MA). Ser-273 Phospho-PPARγ2 antibody was purchased from Bioss Inc. (Bioss, Inc., Woburn, MA).

### Cell culture and differentiation assays

Murine marrow-derived U-33 and AD2 cells represent clonal cell lines spontaneously immortalized in the long term bone marrow culture [2]. To study the effect of PPARγ agonists on marrow MSC differentiation, U-33 cells were stably transfected with either pEF-PPARγ2 expression construct (referred to as U-33/γ2 cells) or an empty vector pEF-BOS (referred to as U-33/c cells), as described previously [2]. In the pEF-BOS expression vector, the coding sequence of interest is under the control of the promoter for human translation elongation factor EF-1α, which permits the levels of ectopically expressed transcript to be at the physiological range [2], [30]. U-33/γ2 and U-33/c cells were maintained in MEM-α supplemented with 10% FBS, 1% penicillin/streptomycin solution, and 0.5 mg/ml G418 for positive selection of transfected cells. The effect of tested compounds on alkaline phosphatase activity, fat accumulation, and gene expression was measured after 3 days treatment, according to previously described protocols [31]. To measure the effect on proliferation, cells were seeded at the density of 3×10^3^ cells/cm^2^ on 96 well plates. After 24 h of growth, cultures were treated with different doses of tested compounds for additional 72 h followed by measuring a rate of cell proliferation using Cell Titer 96 AQueous Non-Radioactive Cell Proliferation Assay. Each experiment was repeated three times.

### Gene expression

Relative gene expression was analyzed using quantitative real time PCR, as described [32]. Briefly, total RNA was extracted using RNeasy Mini kit (Qiagen, Germantown, MD). Its purity and concentration were determined using Agilent 2100 Bioanalyzer (Agilent Technologies, Santa Clara, CA). After DNase treatment, 0.75 µg of RNA was converted to cDNA using the iScript cDNA synthesis kit. The amount of cDNA corresponding to 7.5 ng of RNA was used for each reaction containing Power SYBR Green mix and was processed using StepOne Plus System (Applied Biosystems, Carlsbad, CA). Relative gene expression was determined by the ΔΔ-Ct method using 18S RNA levels for sample-to-sample normalization and using StepOne Plus System software. Primers were designed using Primer Express 3.0 software (Applied Biosystems).

### Western blot analysis

AD2 cells were treated for 60 min with either 1 µM ROSI or 50 µM TEL followed by isolation of proteins by incubating pelleted cells with the whole cell extract buffer (20 mM HEPES, 25% glycerol, 0.42 M NaCl, 0.2 mM EDTA, pH 7.4) supplemented with protease and phosphatase inhibitors (sodium orthovanadate and sodium fluoride) for 10 min on ice. The samples were centrifuged at 100,000×g for 5 min at 4°C and debris were discarded. Protein samples were resolved by 10% SDS polyacrylamide gel electrophoresis and electrophoretically transferred to Immobilon-FL membranes. Membranes were blocked at room temperature for 1 h in TBS [10 mM Tris-HCl (pH 7.4), 150 mM NaCl] containing 3% BSA plus phosphatase inhibitors followed by overnight incubation with primary antibody at 4°C. After three washes in TBST (TBS plus 0.1% Tween 20), membranes were incubated with infrared anti-rabbit (IRDye 800, green) or anti-mouse (IRDye 680, red) secondary antibodies (LI-COR Biosciences, Lincoln, NE) at 1∶15,000 dilution in TBS for 2 h at 4°C. Immunoreactivity was visualized and quantified by infrared scanning in the Odyssey system (LI-COR Biosciences, Lincoln, NE) and band density was quantified using Image J software.

### Animals and experimental design

Obese diabetic yellow agouti A^vy^/a strain (VY/WffC3Hf/Nctr-A^vy^) and lean non-diabetic a/a strains (VY/WffC3Hf/Nctr-a) were originally developed by Dr. G. Wolff (National Center for Toxicology Research, Jefferson, AR) [33] and maintained at the University of Toledo Health Sciences Campus (Toledo, Ohio). The diabetic phenotype for A^vy^/a mice, which is characterized by hyperglycemia, hyperinsulinemia, glucose intolerance and insulin resistance, is attained due to constitutive expression of agouti protein driven by the LTR of an intracisternal A particle (IAP) inserted in the promoter region of agouti gene [34]. In the hypothalamus, the agouti protein suppresses an activity of melanocortin receptor 4 (MC4R) regulating food intake and energy expenditure [33]. The diabetic phenotype of A^vy^/a males develops at the age of 8 weeks. The expression of agouti protein is naturally suppressed in a/a mice and this strain serves as a non-diabetic control to A^vy^/a strain. Five month old males of yellow agouti A^vy^/a strain were used in these studies. Eight month old C57BL/6 males were fed high fat diet (Product #D12451, Research Diets, Inc., New Brunswick, NJ 0890) for 1 mo to develop DIO and glucose intolerance. Body weight and composition were assessed by NMR, and glucose and insulin tolerance tests were performed for both models at the beginning and at the end of experiment. Mice were housed in a constant temperature on a 12-hour light-dark cycle. All animal treatments and care protocols were approved by the University of Toledo Institutional Animal Care and Use Committee (IACUC).

To establish a dose of TEL which equally to ROSI normalizes glucose tolerance, A^vy^/a mice (n = 4 per group) received either TEL in drinking water at the doses of 1.5 and 3 mg/kg/d, or ROSI (Avandia, GlaxoSmithKline) in chow at the dose of 20 mg/kg/d for 4 days followed with intraperitoneal glucose tolerance test (IGTT) [35], [36]. This dose of ROSI is used as a standard dose in our animal models of bone loss due to PPARγ activation [5], [35]. Mice were fasted for 4 h before an ip injection of 2 mg/kg of glucose. Glucose disposal was measured in the blood derived from tail using AlphaTrack Blood Glucose Meter (Abbott Laboratories Inc., Almeda, CA) at the 0, 30, 60, and 120 min time intervals after glucose injection. Doses of 3 mg/kg/day TEL and 20 mg/kg/d ROSI were chosen for the next experiment as equally normalizing glucose tolerance in A^vy^/a animals.

To test the skeletal effects of TEL administration, A^vy^/a or DIO mice were divided into groups (n = 6–8 per group) and fed for 4 weeks with either non-supplemented chow, or chow supplemented with 20 mg/kg/d ROSI (Mol.mass  = 357.428 g/mol), or either water supplemented with 3 mg/kg/d TEL (Mol.mass = 514.617 g/mol) (Sigma) (A^vy^/a mice) or chow supplemented with TEL (Micardis, Boehringer Ingelheim) at the dose 3 mg/kg/d (DIO mice). In addition, a group of A^vy^/a mice received both ROSI-supplemented chow and TEL-supplemented water in the doses listed above. During the experiment, food and water intake per cage were monitored and the average intake of ROSI and TEL per mouse was calculated. There were no differences between groups within each mouse strain in daily food intake and water intake. Calculated dose of effective drug intake in A^vy^/a mice was 21.6 mg/kg/d for ROSI and 4.8 mg/kg/d for TEL, whereas drug intake in DIO mice corresponded to 16.9 mg/kg/d ROSI and 3.3 mg/kg/d TEL. Mice metabolic activity was assessed at the end of experiment using Comprehensive Laboratory Monitoring System (CLAMS) (Columbus Instruments, Columbus, OH) for indirect calorimetry to measure energy balance. Immediately after euthanasia by cervical dislocation under CO2 anesthesia, blood was collected by cardiac puncture and serum samples were prepared. Serum measurements included: random glucose levels, triglycerides (TGs) levels using the Triglyceride Quantification Kit (BioVision, Inc., Milpitas, CA), bone/liver/kidney specific alkaline phosphatase (BALP) using the Alkaline Phosphatase Diagnostic Kit (Sigma-Aldrich) in the presence of 10 mM L-phenylalanine to exclude intestinal ALP enzymatic activity, and tartrate-resistant acid phosphatase form 5b (TRAP5b) using an ELISA assay provided by Immunodiagnostic Systems Inc. (Scottsdale, AZ).

### mCT analysis of trabecular bone

Trabecular bone parameters of L4 vertebrae were analyzed using micro-computed tomography μCT35 system (SCANCO Medical AG, Bassersdorf, Switzerland) Scans were performed at 70 kV, energy and 113 µA intensity settings and using 7 µm voxel. Images of trabecular bone were segmented at 289 threshold value using per mille scale [5], [37].

### Indentation

Cortical bone material properties were measured in midshaft tibia using a reference probe microindentation method [38]. Five indentation tests per specimen were performed by the manufacturer with the BioDent instrument (Active Life Scientific, Inc., Santa Barbara, CA). Measurements included stiffness (N/µm), total indentation distance (TID, µm), indentation distance increase (IDI, µm), and creep indentation distance (CID, µm).

### Statistical analysis

All experiments, including *in vivo* testing, were performed in duplicates or triplicates. Statistical differences between cell treatment conditions were analyzed using one-way ANOVA with Tukey pairwise comparison for equal variances, whereas statistical differences between animal's treatment groups were analyzed using one-way ANOVA with Dunnett's T3 pairwise comparison test for unequal variances using SPSS software. All data represent means and standard deviation of the means (SD). Statistical significance was set to p<0.05.

## Results

### TEL is a selective PPARγ agonist with a weak lipogenic but strong lipolytic activity in adipocytes and lacking anti-osteoblastic activity

TEL activity as PPARγ agonist and its effect on marrow MSCs differentiation was tested in a model of U-33/γ2 cells where differentiation toward osteoblasts and adipocytes is under control of PPARγ2 isoform. This model has been previously validated as advantageous for testing the activities of different natural and artificial PPARγ ligands including oxidated derivatives of linoleic acid, 15dPGJ_2_, and different TZDs [4], [31], [39].

**Figure 1:**
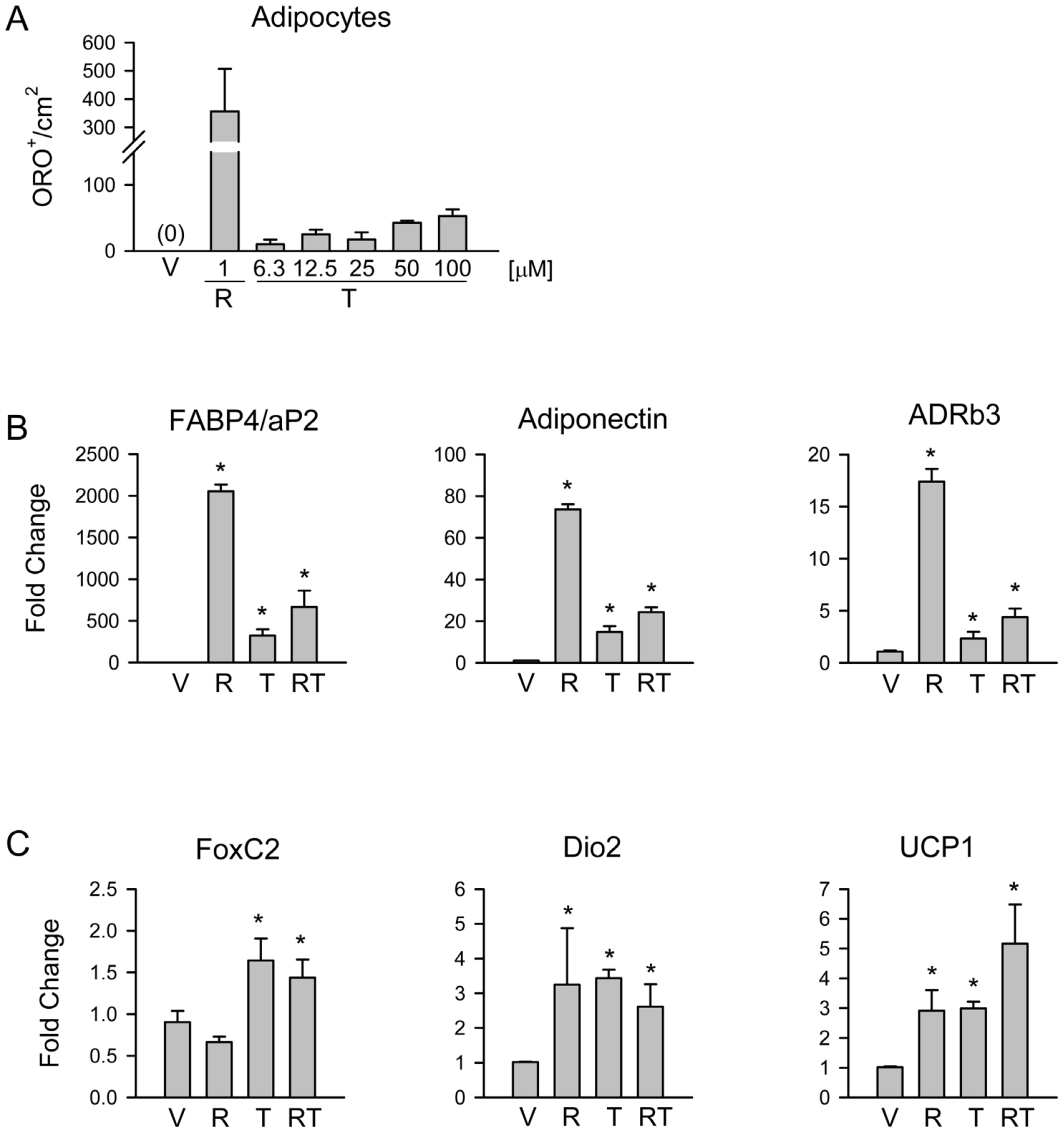
The effect of TEL and ROSI on adipocytic phenotype of U-33/γ2 cells. Cells were treated for 3 days with either vehicle (DMSO) (V), or ROSI (R), or TEL (T), or ROSI and TEL (RT). A. Number of adipocytes in response to treatment with TEL at different concentrations or with 1 µM ROSI and assessed by staining of intracellular lipids with Oil Red O. B. Relative expression of WAT-specific gene markers in response to either 1 µM ROSI, or 50 µM TEL, or 1 µM ROSI and 50 µM TEL. C. Relative expression of BAT-specific gene markers in cells treated as in B. * p<0.05 *vs*. vehicle.

The adipocytic activity of TEL was evaluated as accumulation of lipids and induction of adipocyte-specific gene expression in U-33/γ2 cells and compared to the effect on U-33/c cells which served as a negative control. The effect of TEL on adipocyte differentiation was tested at concentrations ranging from 1 µM to 100 µM and compared to the effect of 1 µM ROSI, a dose which has been demonstrated previously as optimal for inducing pro-adipocytic response in U-33/γ2 cells [31]. Consistent with lower than ROSI binding affinity of TEL (TEL: EC_50_ = 463 nM; ROSI: EC_50_ = 112 nM according to [40]), doses higher than 10 µM appeared to be more effective for fat accumulation; however regardless of a dose TEL never achieved higher than 15% efficiency in inducing fat accumulation as compared to a dose of 1 µM ROSI (Figure 1A). Morphological examination showed that lipid droplets are less numerous and smaller in cells treated with TEL as compared to ROSI (not showed). Consistent with a weaker adipocytic activity, the levels of expression of phenotype-specific gene markers including fatty acids binding protein 4 (FABP4/aP2), adiponectin, and adrenergic receptor β3 (ADRβ3), were proportionally lower in cells treated with 50 µM TEL than in cells treated with 1 µM ROSI (Figure 1B). Combined treatment with TEL and ROSI resulted in an induction of expression of tested gene transcripts at the level of TEL treatment alone.

Since TEL had been previously characterized as a ligand which primarily induces lipolytic rather than lipogenic activity of PPARγ [41], [42], we examined its effect on the expression of genes characteristic for brown adipocytes. Indeed, TEL significantly increased the expression of transcripts specific for transcriptional regulator forkhead box C2 (FoxC2), and proteins involved in lipolysis and energy dissipation such as deiodinase 2 (Dio2) and uncoupling protein 1 (UCP1), respectively (Figure 1C). While induction of conventional adipocyte markers like FABP4/aP2, adiponectin, and ADRβ3, was markedly lower with TEL than with ROSI treatment, the induction of Dio2 and Ucp1 expression was at the same levels in cells treated with either 50 µM TEL or 1 µM ROSI, while expression of FoxC2 was induced exclusively in cells treated with TEL (Figure 1C). This indicates that although TEL is a weak activator of lipogenic PPARγ activity, it has a robust activity towards “beiging” of adipocyte phenotype. Combined treatment with TEL and ROSI showed a gene expression response characteristic for TEL. As expected, the adipocytic response to treatment with TEL and/or ROSI occurred only in U-33/γ2 cells, but not in U-33/c cells (not showed).

**Figure 2:**
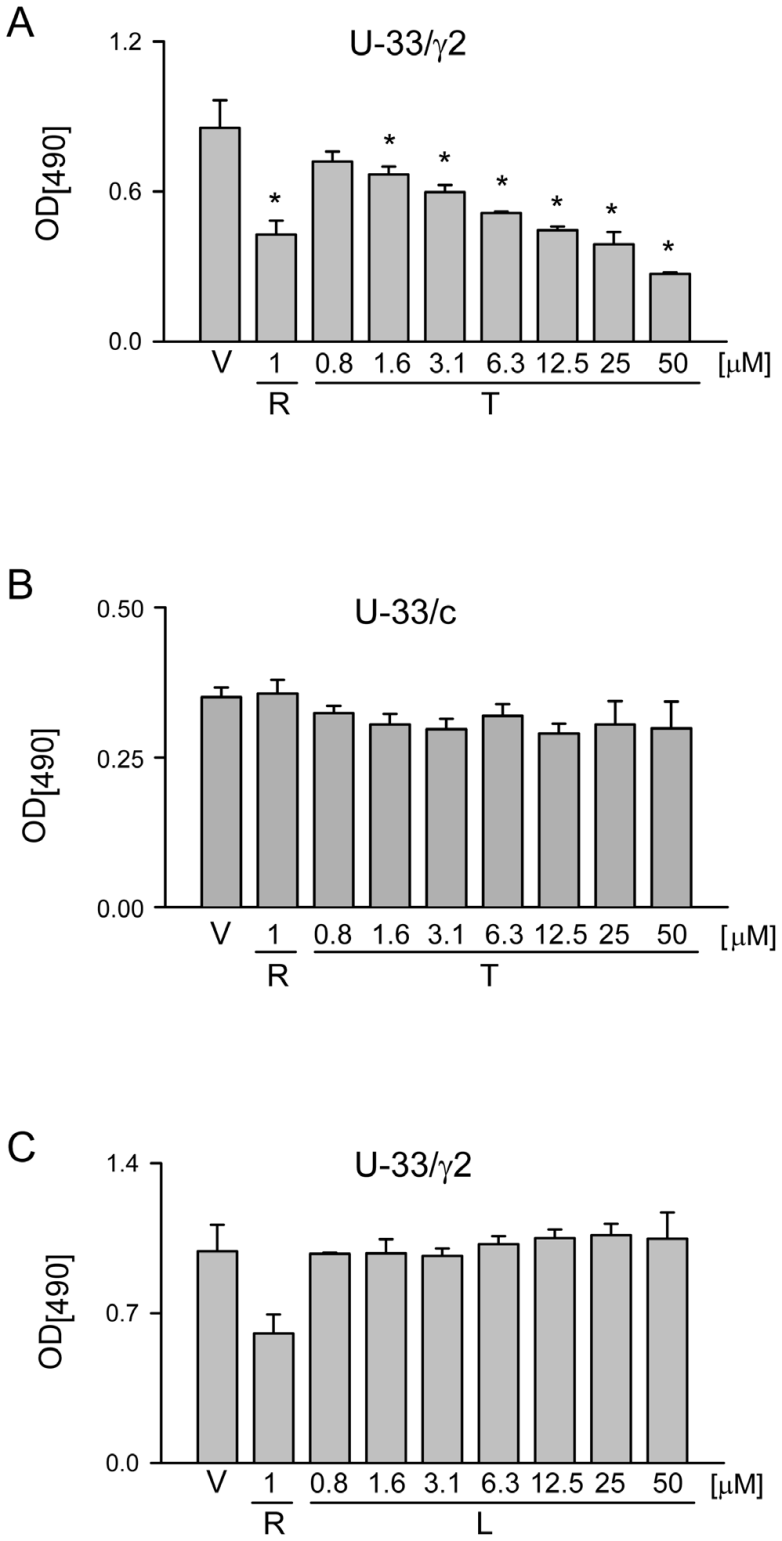
The effect of TEL and losartan (LOS) on proliferation of U-33/γ2 and U-33/c cells. Cell proliferation was assessed using MTT assay after 3 days treatment with tested compounds at different concentrations. A. Dose response of U-33/γ2 cells to treatment with TEL. B. Dose response of U-33/c cells to treatment with TEL. C. Dose response of U-33/γ2 cells to treatment with LOS. V – vehicle; R – ROSI; T – TEL; L – LOS. * p<0.05 *vs*. vehicle.

Further, TEL activity as PPARγ agonist was tested for its effects on cells proliferation [1]. As showed in Figure 2A, TEL inhibited U-33/γ2 cell proliferation in a dose-dependent manner achieving similar effects as 1 µM ROSI at the concentrations higher than 10 µM. The anti-proliferative effect of TEL was not seen in U-33/c cells (Figure 2B). To confirm that TEL anti-proliferative and pro-adipocytic activities require activation of PPARγ2, we tested the response of U-33/γ2 cells to treatment with losartan, another ARB which does not bind nor activate PPARγ. Losartan did not inhibit proliferation of U-33/γ2 cells (Figure 2C), nor induced fat accumulation, nor affected the expression of adipocyte-specific gene markers (not shown). These together indicate that the pro-adipocytic and the anti-proliferative effects of TEL result from direct activation of PPARγ2 protein in U-33/γ2 cells.

**Figure 3:**
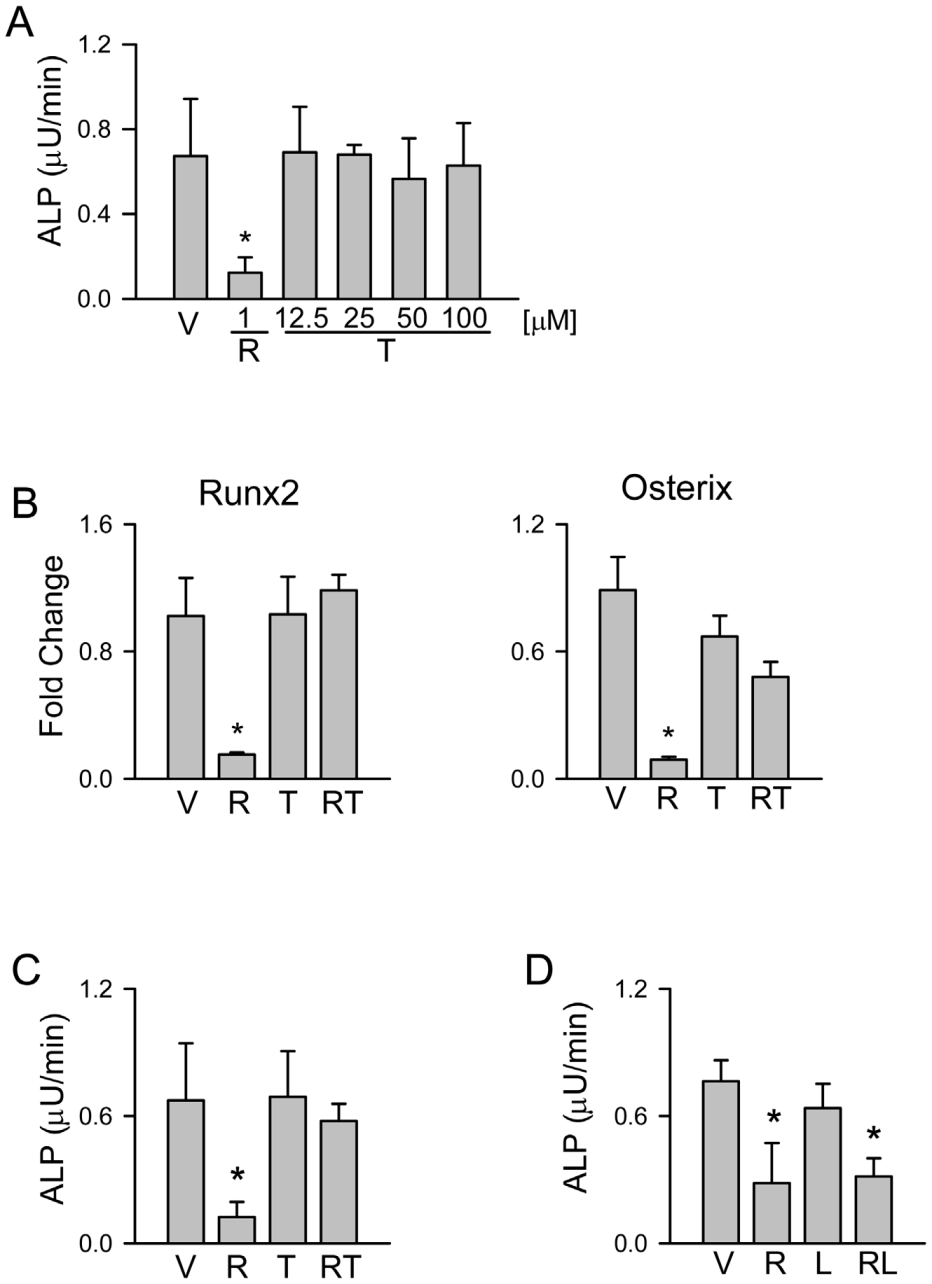
The effect of TEL, LOS, and ROSI on osteoblastic phenotype of U-33/γ2 cells. Cells were treated for 3 days with either vehicle (DMSO) (V), or ROSI (R), or TEL (T), or LOS (L), or in combination (RT or RL). A. Enzymatic activity of alkaline phosphatase (ALP) after treatment with different doses of TEL or 1 µM ROSI. B. Relative expression of osteoblast-specific transcription factors, Runx2 and Osterix, in cells treated with either 1 µM ROSI, or 50 µM TEL, or in combination. C. ALP activity in cells treated with either 1 µM ROSI, or 50 µM TEL, or in combination. D. ALP activity in cells treated with either 1 µM ROSI, or 50 µM LOS, or combination. ALP activity was normalized to the number of cells assessed in MTT proliferation assay (panels A, C, and D). * p<0.05 *vs*. vehicle.

We have shown previously that ROSI induces anti-osteoblastic activity of PPARγ2 [2], [4], and that this activity can be obliterated by either using ligands of different chemical structures or by modifying PPARγ2 amino acid composition in the ligand binding domain [30], [31], [39]. A pattern of induction of PPARγ2 anti-osteoblastic activity revealed that TEL not only acts as a selective agonist lacking the anti-osteoblastic activity, but also as an antagonist being able to compete with ROSI anti-osteoblastic activity (Figure 3). Treatment of U-33/γ2 cells with different doses of TEL did not inhibit alkaline phosphatase (ALP) activity (Figure 3A) and did not suppress the expression of two transcription factors essential for osteoblast differentiation, Runx2 and Osterix (Figure 3B). In cells treated simultaneously with both drugs, TEL counteracted the suppressive effect of ROSI on both Runx2 and Osterix expression (Figure 3B) and on ALP activity (Figure 3C). To test whether the sparring effect of TEL on ROSI anti-osteoblastic activity is mediated through PPARγ, the ALP activity was measured in U-33/γ2 cells simultaneously treated with ROSI and losartan. As showed in Figure 3D, losartan did not protect from ROSI-induced decrease in ALP activity indicating that TEL antagonizes ROSI anti-osteoblastic effect *via* direct interaction with PPARγ.

### TEL does not affect activity of pro-osteoblastic TGFβ/BMP signaling and antagonizes ROSI negative effect on this pathway

**Figure 4:**
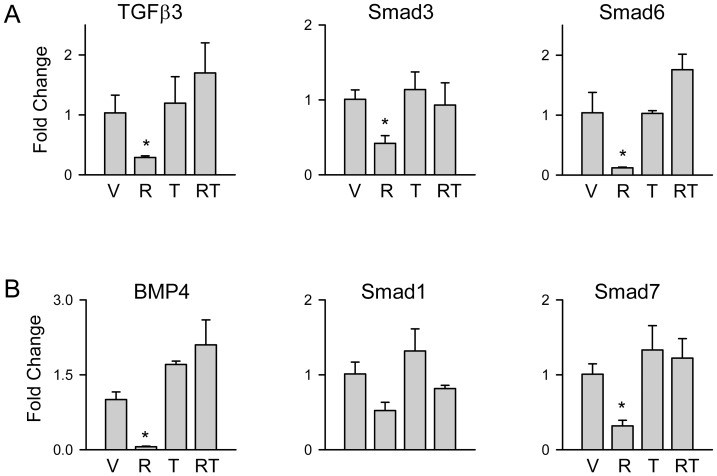
TEL effect on expression of members of TGFβ (A) and BMP (B) signaling pathways. U-33/γ2 cells were treated for 3 days with either vehicle (DMSO) (V), or 1 µM ROSI (R), or 50 µM TEL (T), or in combination (RT). * p<0.05 *vs*. vehicle.

Since we have showed previously that ROSI suppressive effect on osteoblast phenotype is mediated by suppression of several osteoblast-specific signaling pathways including TGFβ/BMP [4] and the evidence that TEL decreases TGFβ activity in tissues other than bone [43], we have analyzed its effect on the expression of members of this pathway which had been identified as suppressed in marrow MSC with ROSI treatment [4]. In contrast to ROSI, TEL did not affect the expression of TGFβ3 and BMP4 growth factors, nor SMAD3 and SMAD1 intracellular mediators, nor SMAD6 and SMAD7 pathway inhibitors, which are under positive regulation of TGFβ/BMP signaling (Figure 4A and B). This pattern of expression suggests that the activity of TGFβ/BMP signaling is preserved in bone marrow cells of mesenchymal origin treated with TEL. Consistent with an antagonistic effect of TEL on ROSI anti-osteoblastic activity, TEL prevented ROSI-induced suppression of all tested members of TGFβ/BMP pathway, except Smad1 (Figure 4A and B).

### PPARγ activation with TEL maintains Ser112 phosphorylation while decreases phosphorylation of Ser273

**Figure 5:**
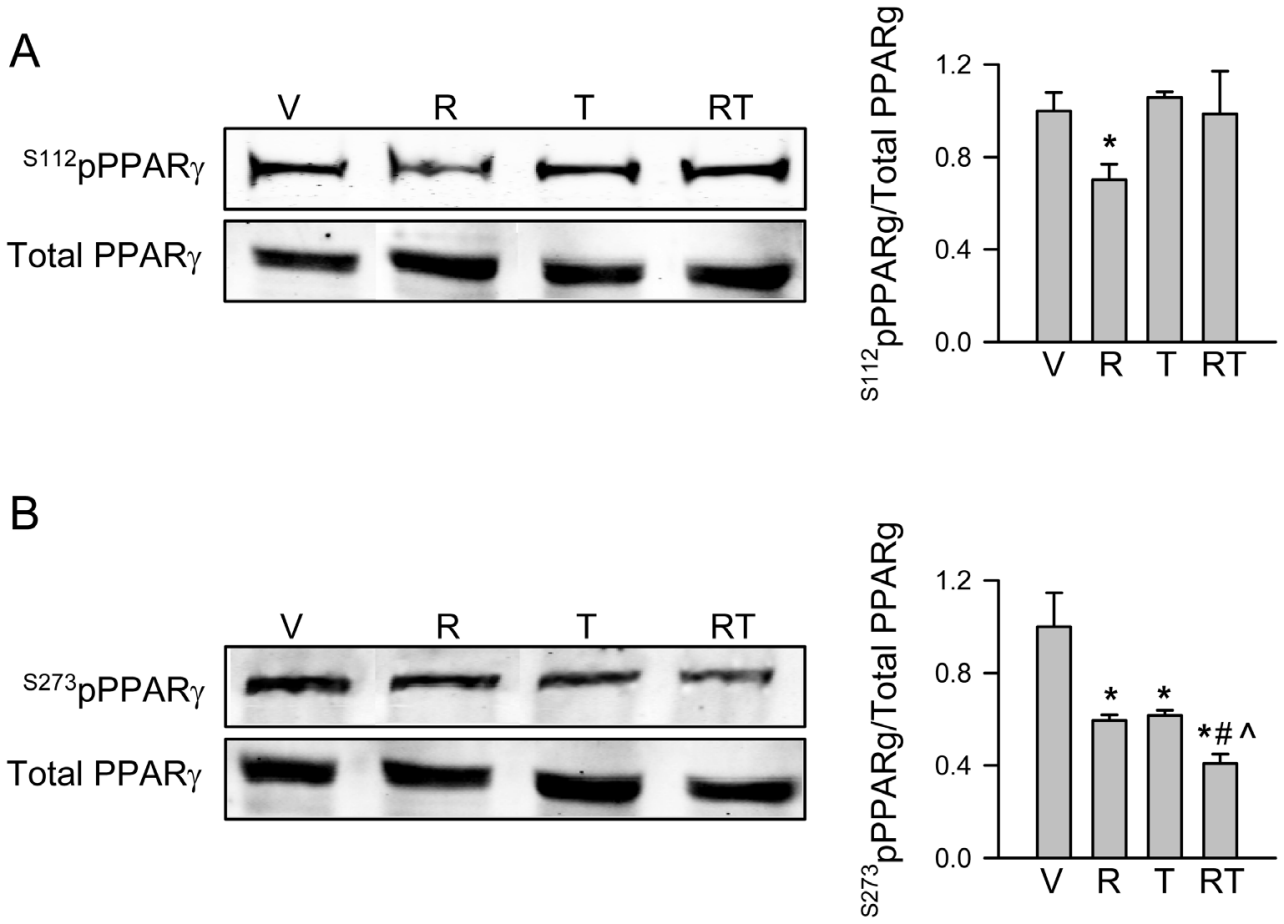
Western blot analysis of ^S112^pPPARγ (A) and ^S273^pPPARγ (B) protein levels after treatment for 60 min with either vehicle (DMSO) (V), or 1 µM ROSI (R), or 50 µM TEL (T), or in combination (RT). * p<0.05 *vs*. vehicle, # p<0.05 vs. ROSI, ∧ p<0.05 vs. TEL.

Treatment with TEL did not affect relative levels of PPARγ phosphorylated at Ser112, whereas ROSI significantly decreased these levels (Figure 5A). This is consistent with TEL weak pro-adipocytic activity, since Ser112 dephosphorylation is required for PPARγ transcriptional activity toward lipid accumulation [13]. Moreover, TEL blocked ROSI-induced decrease in Ser112 phosphorylation, which correlated with an altering of pro-adipocytic and blocking of anti-osteoblastic activities of PPARγ (Figure 1 and Figure 3). Both drugs however, whether applied alone or in combination, decreased phosphorylation of Ser273, an essential step for acquiring insulin-sensitizing activity by PPARγ (Figure 5B).

In summary, presented *in vitro* analyzes indicate that although TEL possesses a weak pro-adipocytic activity toward lipid accumulation, it selectively activates “beige” adipocyte gene expression, and in contrast to ROSI it does not induce anti-osteoblastic activity typified by suppression of lineage-specific markers and TGFβ/BMP signaling. These activities of TEL require interaction with PPARγ2, because U-33/c cells did not respond to TEL treatment and TEL, but not LOS which does not bind to PPARγ, antagonized ROSI pro-adipocytic and anti-osteoblastic activities in U-33/γ2 cells. An altered pro-adipocytic activity and a lack of anti-osteoblastic activity of TEL are associated with an absence of ^Ser112^pPPARγ dephosphorylation.

### TEL administration at the dose which normalizes glucose disposal in murine models of hyperglycemia and glucose intolerance does not affect bone mass

**Figure 6:**
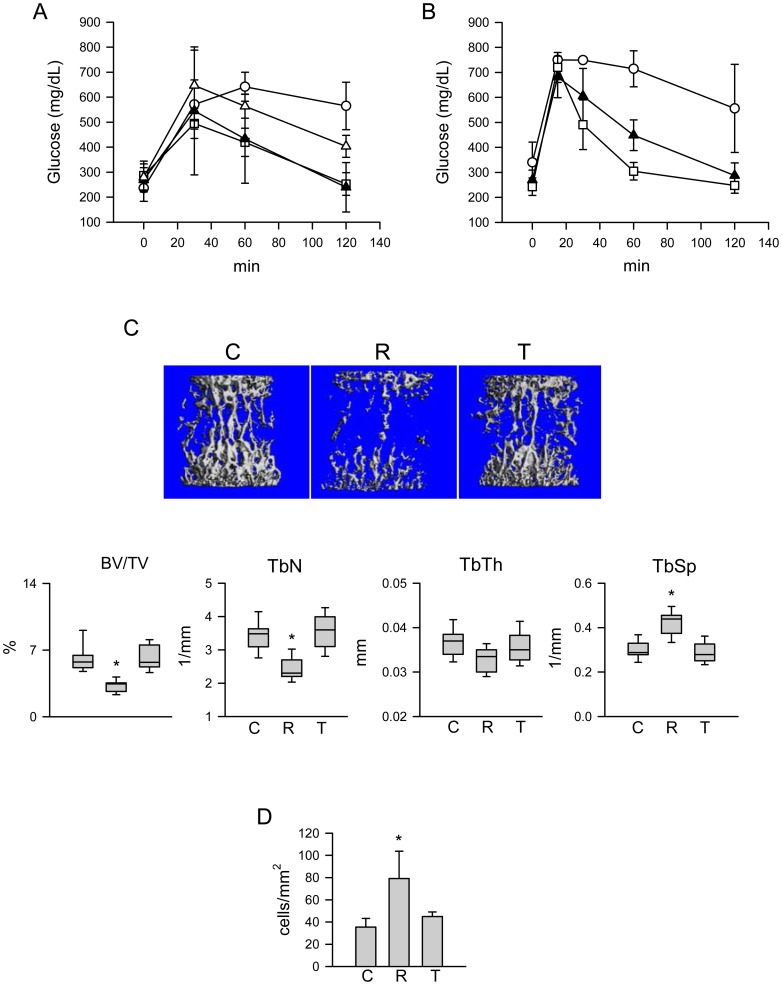
TEL effect on glucose disposal and bone structure. A. Effect of 4 days administration of either regular diet (open circles), or diet supplemented with 1.5 mg/kg/d TEL (open triangles), or 3 mg/kg/d TEL (black triangles), or 20 mg/kg/d ROSI (open squares), on glucose tolerance of A^vy^/a mice measured with introperitoneal glucose tolerance test (IGTT), as described in Material and Methods (n = 4 animals per group). B. Glucose disposal measured with IGTT in DIO mice at the end of 4 wks administration of either non-supplemented HFD (open circles), or HFD supplemented with 3 mg/kg/d TEL (black triangles), or with 20 mg/kg/d ROSI (open squares) (n = 8 animals per group). C. mCT analysis of L4 vertebra trabecular bone of A^vy^/a mice after 4 wks administration of either control non-supplemented diet (C), or chow supplemented with 20 mg/kg/d ROSI (R), or drinking water supplemented with 3 mg/kg/d TEL (T). BV/TV – bone volume fraction in the region of interest (ROI) (%); Tb.N. – average number of trabeculae per unit length (1/mm) of ROI; Tb.Th. – trabecular thickness (mm); Tb.Sp. – trabecular separation representing mean distance between trabeculae (mm). D. Number of adipocytes in proximal tibia of experimental animals (n = 4 per group). C – control; R – ROSI; T – TEL. * p<0.05 *vs*. control.

To assess TEL effect on bone at the dose equal for its anti-diabetic effect to the dose of ROSI, which was previously determined as causing substantial decrease in trabecular bone mass in both normoglycemic and hyperglycemic mice [5], [9], we used two models of impaired murine energy metabolism, yellow agouti A^vy^/a mice and C57BL/6 mice with DIO. A^vy^/a mice develop hyperglycemia, hyperinsulinemia, insulin resistance, and obesity due to suppression of αMSH signaling in the hypothalamus, whereas C57BL/6 mice develop obesity and hyperglycemia in response to feeding with high fat diet (HFD). Initially, two doses of TEL, 1.5 and 3 mg/kg/d, were compared to the 20 mg/kg/d of ROSI for their efficacy to normalize glucose disposal. As showed in Figure 6A, administration of TEL at a dose of 3 mg/kg/d for 4 days improved glucose tolerance in A^vy^/a mice to the degree comparable to the 20 mg/kg/d dose of ROSI, whereas a dose of 1.5 mg/kg/d TEL was less effective. Subsequently, the dose of 3 mg/kg/d TEL administered for 4 weeks was tested for the effects on bone mass of A^vy^/a and DIO mice, and was compared to a 20 mg/kg/d dose of ROSI. Figure 6B confirms that DIO mice responded to these treatments similarly as A^vy^/a mice in respect to normalization of glucose disposal.

Four weeks of TEL administration, did not affect volume and structure of trabecular bone in vertebra of A^vy^/a and DIO mice (Figure 6C and Table 1). While administration of ROSI resulted in a loss of vertebral trabecular bone volume by approximately 50%, a decrease in trabecular connectivity by 75%, a decrease in a number of trabeculae by 30%, and an increase in spacing between trabeculae by 33% (p<0.001 in all cases), none of these changes were observed in A^vy^/a and DIO animals receiving TEL (Figure 6C and Table 1). Similarly, ROSI induced fat accumulation in the marrow, while TEL did not have an effect on this parameter (Figure 6D).

**Table 1:** Micro-computed tomography (mCT) analysis of trabecular bone in L4 vertebra.

Variable	A^vy^/a mice				DIO mice		
	Control	ROSI	TEL	TEL + ROSI	Control	ROSI	TEL
**BV/TV (%)**	6.17±1.69	3.23±0.70^a^	6.12±1.36^b^	4.95±0.61^b^	9.60±1.40	5.27±0.96^a^	8.48±0.67^b^
**TbN (1/mm)**	3.43±0.51	2.44±0.37^a^	3.54±0.55^b^	2.73±0.17^b^	4.46±0.33	3.67±0.51^a^	4.15±0.17
**TbSp (mm)**	0.300±0.046	0.421±0.060^a^	0.290±0.049^b^	0.372±0.023^a^	0.224±0.017	0.278±0.045	0.241±0.010
**TbTh (mm)**	0.033±0.004	0.033±0.003^a^	0.036±0.001	0.038±0.001^a^	0.040±0.002	0.031±0.001^a^	0.034±0.002^b^
**ConnD (1/mm^3^)**	78.6±35.1	20.1±15.2^a^	71.9±26.9^b^	54.1±5.8^b^	185.7±39.4	82.5±37.7^a^	166.8±30.2^b^

BV/TV - Bone volume fraction; Tb.N. - trabecular number; Tb.Sp. - trabecular separation; Tb.Th. - trabecular thickness; Conn.D - connectivity density. N = 6–8 mice per group. ^a^p<0.05 *vs*. Control; ^b^p<0.05 *vs*. ROSI.

An analysis of the levels of bone turnover markers in sera of TEL treated animals suggested an absence of the effect on bone formation and a negative effect on bone resorption (Table 2). Serum levels of bone formation marker BALP, which showed a tendency for a decrease with ROSI treatment, were not affected in A^vy^/a animals receiving TEL. In contrast, serum levels of bone resorption marker TRAP5b, which had a tendency to increase with ROSI treatment, were significantly decreased in animals treated with TEL consistent with previously reported antiresorptive effect of ARB class of drugs, which is independent of PPARγ activity and involves blocking of Ang II signaling in osteoclasts and osteoblasts [18], [21].

**Table 2:** Bone turnover markers in sera of A^vy^/a mice treated with either TEL, or ROSI, or both drugs.

Marker	Control	ROSI	TEL	TEL + ROSI
BALP (mU/min)	3.5±1.6	1.7±0.2	3.4±0.3	3.6±1.1
TRAP5b (U/L)	2.2±0.2	2.9±0.5	1.7±0.2^a^	2.7±0.4

BALP – bone-specific alkaline phosphatase; TRAP5b – tartrate-resistant acid phosphatase 5b isoform; ^a^p<0.01 *vs*. control.

TEL did not affect material properties of the cortical bone as measured by microindentation method (Table 3). Bone strength and stiffness measured as a depth of penetration of cortical bone with a microindentation probe did not differ between A^vy^/a animals receiving TEL and non-supplemented with a drug diet. In contrast, cortical bone of animals receiving ROSI-supplemented diet showed a 48% decrease in resistance to the force applied, which corresponded to 6% decrease in bone stiffness (Table 3).

**Table 3:** Bone tissue material properties measured by reference probe microindentation method.

Measurement	Control	ROSI	TEL
IDI (µm)	7.76±1.98	11.17±0.88^ab^	8.31±1.41^b^
TID (µm)	44.18±7.86	46.09±12.04	36.92±5.66
CID (µm)	4.15±0.62	5.07±0.09	4.20±0.08^b^
Stiffness (N/µm)	0.12±0.01	0.11±0.01^a^	0.13±0.01^b^

IDI – indentation distance increase; TID – total indentation distance; CID – creep indentation distance; ^a^p<0.05 *vs*. control; ^b^p<0.05 *vs*. ROSI.

To test whether TEL may antagonize ROSI anti-osteoblastic activity *in vivo*, A^vy^/a animals received both TEL and ROSI at the doses of 3 mg/kg/d and 20 mg/kg/d, respectively. As presented in Table 1 and Table 2, TEL partially protected against the bone loss caused by ROSI and restored levels of BALP in sera confirming our *in vitro* observation that TEL blocks ROSI-induced anti-osteoblastic activity of PPARγ.

The effects of TEL and ROSI on metabolic parameters of both mouse models indicate that although both drugs have similar effect on glucose metabolism, they differ in their effects on fat metabolism (Table 4). Both drugs had the same effect on normalization of random glucose and triglyceride levels in serum, and were equally efficacious for induction of expression of genes responsible for glucose metabolism in the liver including glucose 6-phosphatase (Glc6ase), phosphoenolpyruvate carboxykinase (PEPCK), and forkhead box protein O1 (FoxO1) (Figure S1). However, and in contrast to ROSI, TEL did not increase body weight in A^vy^/a mice and prevented a gain of body weight in DIO animals fed HFD (Table 4). In addition, the expression of fatty acids synthase (FAS) was elevated exclusively in the liver of ROSI- but not TEL-treated animals. These together suggest that both drugs differ in their effect on fatty acids metabolism.

**Table 4:** Effect of ROSI and TEL administration on metabolic parameters of A^vy^/a and DIO mice.

Parameter	A^vy^/a mice			DIO mice		
	Control	ROSI	TEL	Control	ROSI	TEL
BW (%)	3.5±1.2	9.5±2.1^a^	4.9±2.5^b^	12.5±6.0	15.9±4.3	1.2±5.8^a,b^
eWAT (g)	1.54±0.25	1.79±0.20	1.47±0.38	1.78±0.33	2.89±0.49^a^	2.17±0.30^b^
iBAT (g)	0.112±0.019	0.226±0.055^a^	0.130±0.024^b^	0.640±0.089	0.405±0.158^a^	0.818±0.096^a,b^
RG (mg/dL)	325±79	212±25^a^	249±45^a^	340±81	243±35	270±39
TG (mg/dL)	178±33	150±32	139±24^a^	ND	ND	ND

BW – change in body weight from the beginning of treatment; eWAT – weight of epidydimal fat at the end of treatment; iBAT - weight of interscapular fat at the end of treatment; RG – serum random glucose levels at the end of treatment; TG – serum triglycerides levels at the end of treatment. ^a^ - p<0.05 *vs* control; ^b^ - p<0.05 *vs* ROSI.

**Figure 7:**
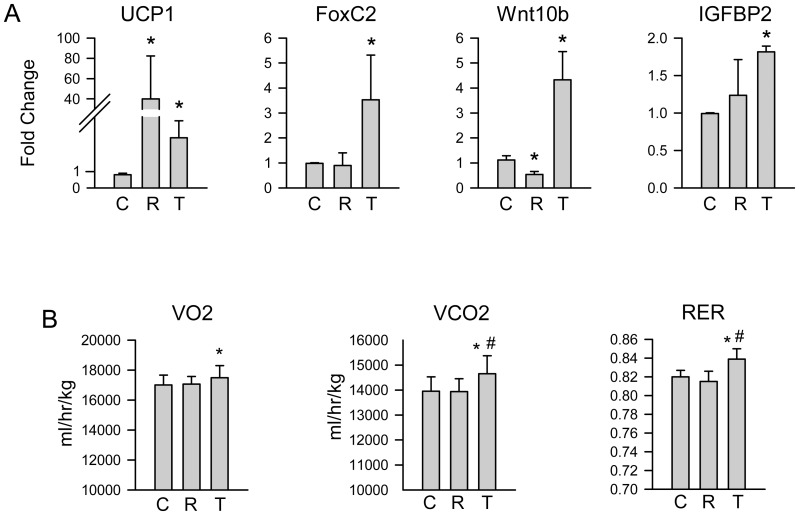
TEL effect on energy metabolism parameters. A. Expression of metabolic gene markers in eWAT. B. Respiratory parameters of DIO mice after 4 wks of treatment measured in CLAMS metabolic cages during a dark day cycle (12 h). C – control; R – ROSI; T – TEL. *p<0.05 vs. control.

As showed in Figure 7A, TEL induced “beiging” of eWAT. The expression of beige fat gene markers, including UCP1 and FoxC2, was significantly increased in animals receiving TEL. Interestingly, “beiging” of eWAT correlated with increased expression of Wnt10b and IGFBP2, two endocrine/paracrine factors recognized for their anti-obesity and bone anabolic activity [44]–[46]. Consistent with prevention in body weight increase and with “beiging” of eWAT, DIO animals receiving TEL had increased oxygen consumption and carbon dioxide production, which resulted in increased respiration rate (Figure 7B). These results confirm observations by others that TEL increases energy expenditure [41], [42].

## Discussion

Presented studies demonstrate that activation of PPARγ with a partial agonist TEL, at the dose which improves glucose tolerance in diabetic animals with similar efficacy as full agonist ROSI, does not affect bone mass. Most importantly, we have shown that the lack of TEL anti-osteoblastic effect is a result of an active interaction with PPARγ protein. *In vitro*, TEL antagonized ROSI anti-osteoblastic effect including suppression of phenotype-specific gene expression and an activity of TGFβ/BMP signaling pathway. *In vivo*, TEL provided a partial protection from ROSI induced bone loss. While having an opposite to ROSI effect on PPARγ activities regulating osteoblast differentiation and function, TEL had a similar effects on regulation of glucose metabolism and insulin sensitivity, indicating that these two PPARγ activities can be regulated independently. This finding is of importance considering an ongoing quest for new insulin sensitizers and PPARγ agonists which have beneficial anti-diabetic activities without undesired and deleterious effect on bone of classical TZDs.

ROSI negative effect on bone results from unbalanced bone remodeling with decreased osteoblastogenesis and increased osteoclastogenesis in part due to increased RANKL production by osteoblast [5]. In presented studies we focused on the TEL effect on osteoblastic activity of PPARγ. By using an *in vitro* model of MSC differentiation under PPARγ2 control, we have identified TEL activities which are conveyed through PPARγ and are independent of Ang II signaling. The TEL anti-proliferative activity and induction of beige fat phenotype in mesenchymal cells are mediated through PPARγ, as well as TEL activity to antagonize the negative effect of ROSI on both osteoblast phenotype and TGFβ/BMP signaling pathway. The TGFβ/BMP signaling is essential for regulation of bone acquisition and bone remodeling, mainly through the regulation of marrow MSC lineage commitment and osteoblast maturation [47], and its activity is compromised with aging [48], in osteoporotic patients [49], and in patients on TZD therapy [50]. All these conditions are associated with increased lipogenic, pro-adipocytic activity of PPARγ in marrow cells of mesenchymal lineage.

Different activities of PPARγ are determined by its interaction with specific ligand and assembly of cofactors on the PPARγ/RXR protein complex. As compared to ROSI, the unique mode of PPARγ activation with TEL results from its differential binding to the ligand pocket, which leads to a recruitment of an altered set of cofactors including coactivators such as SRC1, GRIP, PGC1α and PGC1β, and corepressors SMRT and NCoR [28], [51]. The specificity of cofactors assembly is driven by postranscriptional modification of PPARγ protein including phosphorylation of Ser112 and Ser273 [52], [53]. We have shown that both TEL and ROSI have the same effect on dephosphorylation of Ser273, which determines insulin-sensitizing activity, but differ in their effect on dephosphorylation of Ser112, which is essential for acquisition of transcriptional adipocytic activity toward lipid accumulation [12], [13]. We have shown that in bone marrow MSCs high levels of ^Ser112^pPPARγ upon TEL treatment correlated with acquisition of pro-beige fat and an absence of anti-osteoblastic activity of PPARγ, whereas low levels of ^Ser112^pPPARγ upon ROSI treatment correlated with lipid accumulating pro-adipocytic and anti-osteoblastic activities of PPARγ. At this point however we cannot conclude about the role of Ser112 in regulation of anti-osteoblastic activity of PPARγ, although our preliminary characteristic of mice deficient in PP5 phosphatase, which is responsible for dephosphorylation of Ser112, indicate its involvement in the regulation of marrow MSCs lineage commitment and bone mass (not published).

TEL has been previously recognized for its fat burning activity associated with activation of BAT-specific gene expression. Indeed, TEL induces UCP1 expression in brown adipose tissue and increases oxygen consumption in obese mice [41]. It also increases expression of Sirt1 in WAT, which activates PPARγ fat “beiging” properties resulting from lysine deacetylation [42], [52]. We have showed that an increase in metabolism of DIO mice measured by rate of respiration is associated with “beiging” of WAT. Although we did not examine the effect of TEL on a status of deacetylation of lysine residues in PPARγ protein, our data suggest that Ser112 can be involved in the process of fat “beiging”. If this single PTM regulates both, energy metabolism and osteoblastogenesis, than one can speculate that there is a positive correlation between improved energy metabolism due to fat “beiging” and bone mass. This may suggest a new pharmacological opportunity for simultaneous control of both energy metabolism and bone mass by targeting an activity of PPARγ protein through its PTMs.

TEL-induced “beiging” of WAT in DIO mice included increased expression of FoxC2, Wnt10b and IGFBP2. We have recently reported that fat which acquires beige phenotype due to adipocyte-specific expression of FoxC2 transcription factor releases endocrine/paracrine activities which are anabolic for bone [54]. We have identified IGFBP2 and Wnt10b as being secreted from FoxC2-induced beige adipocytes and being able to activate, in the endocrine manner, osteoblasts and osteocytes for their function to increase bone formation [54]. Beside their bone anabolic activities, IGFBP2 and Wnt10b are also known for their anti-obesity activities [44]–[46]. Thus, these two proteins represent factors which combine both, improvement in energy metabolism and positive regulation of bone mass. It has been reported that extraskeletal bone formation in either atherosclerotic vessels or in heterotopic bone is associated with a presence of adipocytes with brown phenotype suggesting their positive effect on tissue calcification [55], [56]. Although in the presented studies we did not observe bone anabolic activity of TEL, we cannot exclude that prolonged therapeutic use of TEL may be beneficial for bone by inducing bone anabolic activity in fat cells including marrow adipocytes.

In conclusion, presented studies provide a new insight into the PPARγ pro-adipocytic and anti-osteoblastic activities, and suggest an important role of Ser112 in regulating these activities. TEL may provide a model for development of a novel class of PPARγ activators with beneficial metabolic activities, yet safe for bone. They also suggest that these activities can be pharmacologically separated and individually harnessed by using specifically designed selective PPARγ agonists.

## Supporting Information

Figure S1 **Expression of genes regulating glucose and fatty acids metabolism analyzed in the liver of A^vy^/a mice at the end of 4 wks treatment.** C – control; R – ROSI; T – TEL. * p<0.05.
(DOC)

Checklist S1 **Animal Research Reporting In Vivo Experiments (ARRIVE) checklist.**
(DOC)
