# Supplementary material for: Partial Agonist, Telmisartan, Maintains PPARγ Serine 112 Phosphorylation, and Does Not Affect Osteoblast Differentiation and Bone Mass
Source: PLoS One. 2014 May 8;9(5):e96323. doi: 10.1371/journal.pone.0096323 (PMC4014504; doi:10.1371/journal.pone.0096323)
Supplement: Supplementary file 2 [file pone.0096323.s001.doc]

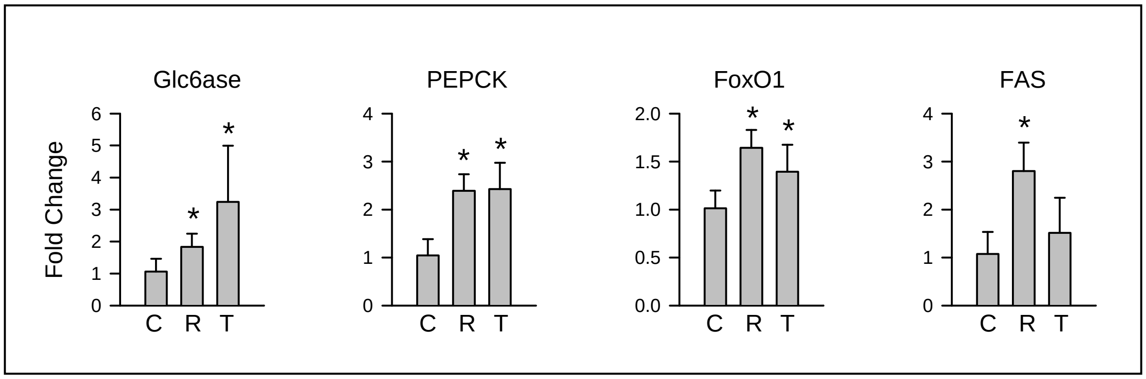
Glc6ase
PEPCK
FoxO1
FAS
Fold Change
0
1
2
3
4
5
6
0.0
0.5
1.0
1.5
2.0
*
C
R
T
